# Controlling bottom-up rapid growth of single crystalline gallium nitride nanowires on silicon

**DOI:** 10.1038/s41598-017-17980-0

**Published:** 2017-12-20

**Authors:** Ko-Li Wu, Yi Chou, Chang-Chou Su, Chih-Chaing Yang, Wei-I. Lee, Yi-Chia Chou

**Affiliations:** Department of Electrophysics, National Chiao Tung Univeristy, Hisnchu, 300 Taiwan

## Abstract

We report single crystalline gallium nitride nanowire growth from Ni and Ni-Au catalysts on silicon using hydride vapor phase epitaxy. The growth takes place rapidly; efficiency in time is higher than the conventional nanowire growth in metal-organic chemical vapor deposition and thin film growth in molecular beam epitaxy. The effects of V/III ratio and carrier gas flow on growth are discussed regarding surface polarity and sticking coefficient of molecules. The nanowires of gallium nitride exhibit excellent crystallinity with smooth and straight morphology and uniform orientation. The growth mechanism follows self-assembly from both catalysts, where Au acts as a protection from etching during growth enabling the growth of ultra-long nanowires. The photoluminescence of such nanowires are adjustable by tuning the growth parameters to achieve blue emission. The practical range of parameters for mass production of such high crystal quality and uniformity of nanowires is suggested.

## Introduction

Nitride materials have been widely used in optoelectronics and high speed electronic devices including light emitting diodes (LEDs)^1–3^, blue laser diode (LD)^4^, and energy harvesting application^5^. Gallium nitride (GaN) has wide and direct band gap for providing wide light spectrum and holds reasonable flexibility of tuning bandgap by implantation such as doping Si, Mg^6^, and Ge^7^. Growth of GaN nanowires, different from typical III-V nanowire growth^8–10^, by the catalytic vapor-liquid-solid (VLS) or vapor-solid-solid (VSS) method is of particular interest^11–15^ owing to the size and one-dimentional structure which are potentially useful in the scaling of nanoelectronics^16,17^, especially in the evolution of current FinFET technology^18,19^. In nanowires, there is less degree of freedom for electron traveling along the radial direction. In addition, quantum confinement might apply and make them quantum wires when the dimension is compatible with the typical wavelength of a conduction electron in bulk, say diameter of a nanowire is less than ~5 nm^20^, yet the surface area of nanowires is larger which benefits to lighting electronics and sensors.

The enhancement in performance of high performing RF devices continues with further CMOS scaling^21^; however, it does not support high-voltage and high-power-density applications required for power amplification and power conversion for scaled CMOS with fine Si dimensions. Alternatively, GaN devices are ideal for high performance power amplifiers operating at frequencies which are unachievable with any Si-only-based technology. GaN power electronics offers lower power loss than Si devices; in addition, they can operate at higher temperatures. Thus, combining strength of both GaN and Si technologies by co-integrating GaN devices with Si integrated circuits provides a scalable CMOS technology platform for high power and high performance optoelectronics.

The cost of Si substrates is lower than sapphire, which is typically used as substrate for growing GaN because of the comparable lattice parameters. GaN has larger lattice mismatch with Si which is the challenge for epitaxial growth of GaN on Si. However, self-assembly of GaN nanowires on Si can possibly overcome the barrier since nanowire is very effective in relaxing the strain and make the epitaxial growth possible^22^.

Here we integrate these benefits and propose the growth of GaN nanowires on Si with high crystal quality and controllable orientation at rapid growth rate via self-assembly. The growth of high crystal quality and high yield GaN nanowires is commonly carried out in molecular beam epitaxy (MBE)^23–26^ and metal-organic chemical vapor deposition (MOCVD)^27–29^, where the substrates are sapphire, but rare in hydride vapor phase epitaxy (HVPE)^30–32^, especially for the growth on Si. The growth in MBE and MOCVD can achieve high quality of 2D and 1D crystals precisely without defects but the growth rate is slow. HVPE is known for efficient growth of high crystalline quality GaN films^33,34^ yet the quality is not as good for growing crystals with high lattice mismatch with substrates. In order to obtain good crystal quality of GaN nanowires with reasonable speed, we use HVPE and apply catalytic reaction in the growth process to allow reaction of the precursors in catalyst. The catalyst mediates the precipitation of the supersaturation of the product on the Si substrate. Thus it directs the growth to achieve high crystal quality at high speed. Besides, Ga forms eutectic liquid with Si which assists the first stage reaction where liquid catalyst gathers molecules.

We report in this article the self-assembly of GaN nanowires with Ni and Ni-Au as catalysts on Si substrates using HVPE. The growth rate is ~330 nm/s and the nanowires are with smooth surface, single crystallinity, straight and consistent in orientation. In other words, we propose the growth of GaN nanowires of a few ten micrometers in length within very short growth time (1 min or 5 min in our experiments). These GaN nanowires are single crystalline with uniform orientation and high density. The growth of ultra-long, e.g. 20 μm, GaN nanowires are potentially useful for the applications in sensors.

## Results and Discussion

### Growth of GaN Nanowires from Metal/Alloy catalysts

Ni and Ni-Au were selected as catalysts where Au acts as a protection from oxidation of Ni and etching during growth by HCl. The orientation, catalyst chemistry, crystallinity, and photoluminescence of the GaN nanowires are investigated and discussed. Furthermore, we discussed the growth mechanism, the effects of V/III ratio and carrier gas flow, thus we propose an optimized range of growth parameters for GaN nanowire growth on Si.

**Figure 1:**
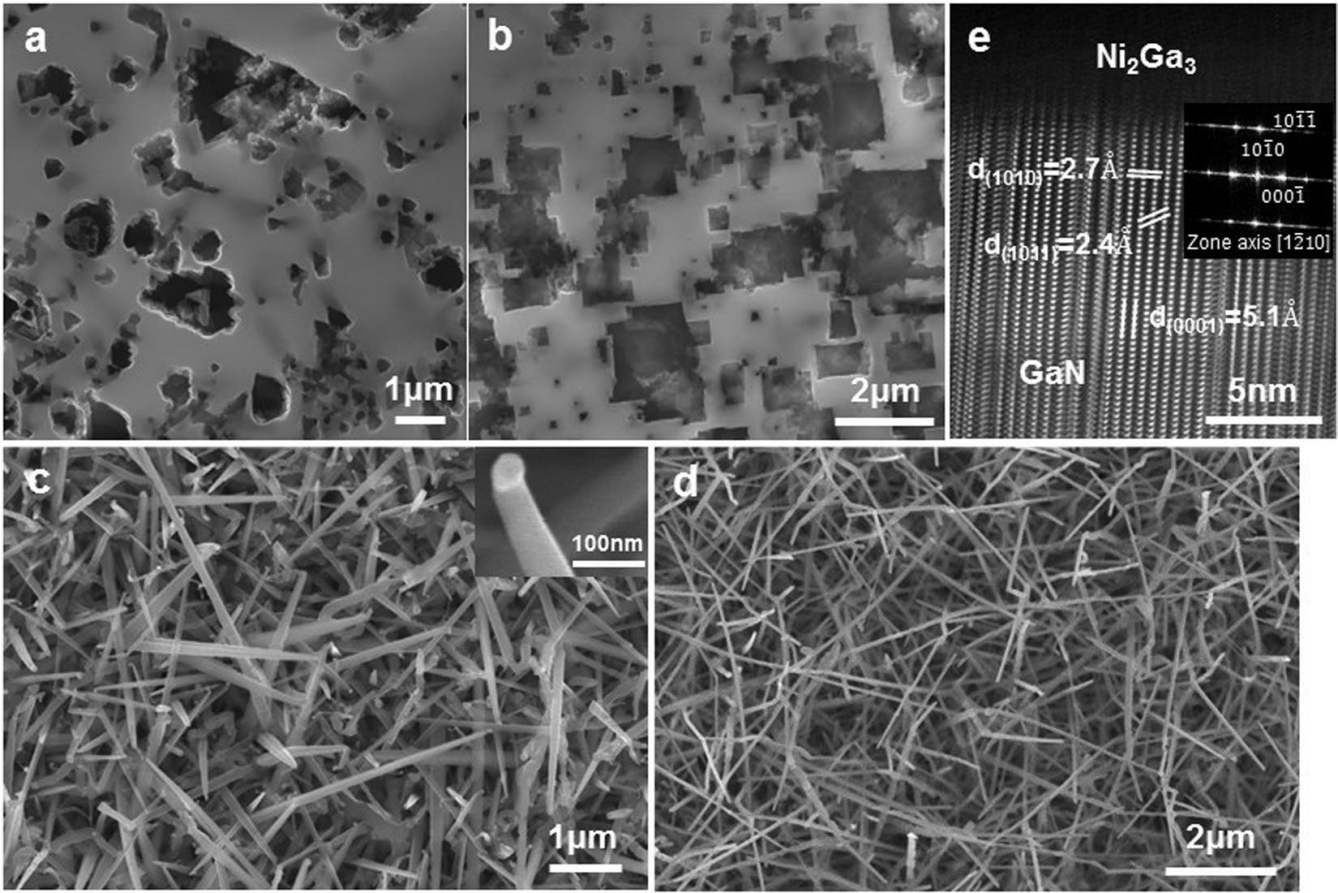
Growth of GaN nanowires on Si (111) and (100) with and without metal catalysts. (**a**,**b**) Growth without catalyst on Si (111) and Si (100), respectively. (**c**) Growth with catalysts of Au-Ni on Si at 880 °C and V/III = 20. The inset shows an enlarge image of a single nanowire with catalyst particle on the tip. (**d**) Growth with catalysts of Ni on Si at 880 °C and V/III = 20. (**e**) HRTEM image of a GaN nanowire in (**c**) along [1210] viewing direction.

The Si substrates without metal deposition were warmed up slowly to 600 °C to allow outgassing and remove the surface contamination, and then treated by high temperature flash before growth to obtain good Si lattice on surface. Without metal catalyst, there were no growth of GaN on neither Si (111) nor (100) substrates, as shown in Fig. 1a and b, respectively, due to the large lattice mismatch between GaN and Si. The surface revealed triangular patterns on Si (111) and square patterns on Si (100) which showed the crystallinity of (111) and (100) surface of Si with diamond structure.

The classic catalyst for VLS growth of Si nanowires is Au, but pure Au is not stable at the general range of growth temperatures (800–1000 °C) of GaN (coarsening of Au cannot be avoided). Thus, Ni was selected as the catalyst for GaN nanowire growth due to the high tolerance to reaction temperature and the low liquidation temperature when mixing with Ga. 1-nm-thick Au was deposited on Ni to protect it from oxidation when handling the sample in air. Figure 1c and d showed the high density GaN nanowires grown at 880 °C on Si (111) from Ni-Au and Ni, respectively. The inset of Fig. 1c showed an enlarged image of a single GaN nanowire. We investigated the structures, crystallinity, and growth orientation of the nanowires, as shown in Fig. 1e, which confirmed the single crystalline wurtzite structure of GaN and the nanowire grew along [1010] direction with m-plane growth front. Note that the stacking faults shown in Fig. 1e were caused by slow cooling when the GaN still grew with residual gases and heat but at inconstant temperature. The effects of cooling step on the nanowire were discussed in ESI.

### Nanowire Growth Mechanism

The growth of GaN had precursor gases of Ga and N involved so that V/III ratios (the ratio of N and Ga) and the amounts of carrier gas flow were of interests on controlling the density and morphology of GaN nanowires. The V/III ratios determined the relative amount of supply and the carrier gas flow determined the flow rate of supply gases and the width of boundary layer which is related to reaction rate. The Si (111) substrates were used in this section.

**Figure 2:**
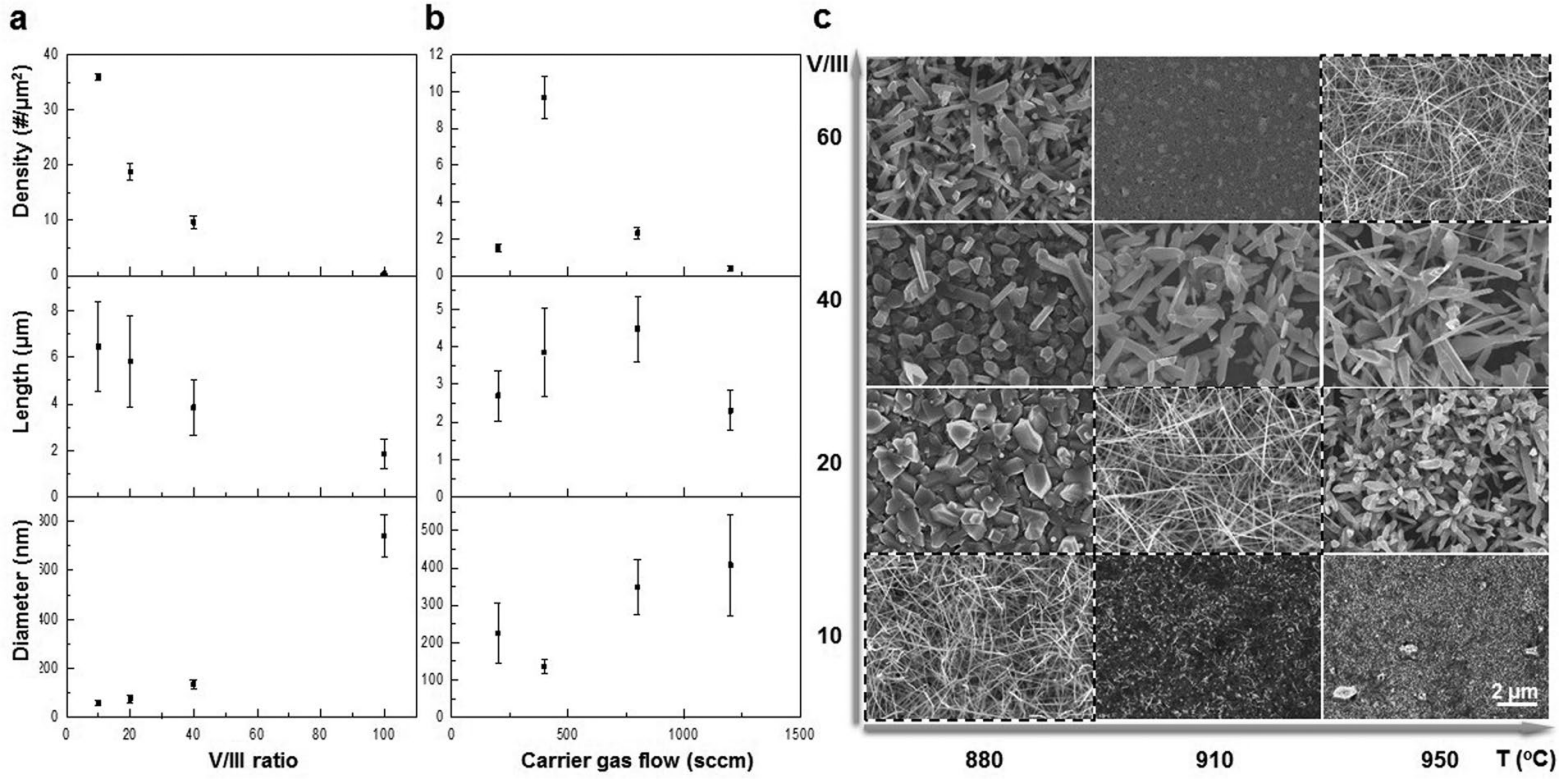
Effects of V/III and carrier gas flow on the growth of GaN nanowires on Si (111). (**a**) The density, length, and diameter of nanowires grown at 880 °C with carrier gas flow of 400 sccm at different V/III ratios. The growth rate for successful nanowire growth is ~107 nm/s. (**b**) The density, length, and diameter of nanowires grown at 880 °C with V/III ratio of 40 at different carrier gas flow. The growth rate for successful nanowire growth is ~65 nm/s. (**c**) SEM images of GaN nanowires grown at different T and V/III ratio under carrier gas flow of 300 sccm. The best grown nanowires at each growth temperature are highlighted by the dashed frames.

Figure 2a and b showed with different V/III ratios (Fig. 2a) and amounts of carrier gas flow (Fig. 2b) at 880 °C. The density and morphology change of the nanowires also depend on growth temperature as shown in Figure S5 where the nanowires exhibit nanowire form at 880 °C. When V/III ratio increased, changing from 10 to 40, the density of the GaN nanostructures decreased, the length of them shrank, and the diameter increased (Note: The length of the nanowires was measured and took average from cross sectional SEM images, where we assume that the total measured period for growth (5 min) is the same with growth period (neglect nucleation period) due to the high precursor pressures). It was because, when the environment became N-rich, it preferred forming N-polarity planes (the plane of 1 $\bar{1}\,$01 or 11 $\bar{2}\,$2)^35^, so the lateral growth of the nanowires dominated instead of growing along the axial direction of nanowires (a-plane or m-plane; will be shown below). It was caused by the adjustment of surface energy and density of dangling bonds by the N-rich environment, where planes with N-polarity and with less density of dangling bonds were stable surfaces^35^. Alternatively, with lower V/III ratio, Ga-polarity dominated where c-plane was stable so that the growth front was along a-plane or m-plane, see example later. The V/III ratio could modify the surface polarity thus changes the growth orientation.

In addition, if increasing V/III ratio to an extreme, e.g. 100, the supply of Ga was too little to reach supersaturation in the catalyst for nucleation and growth of GaN. Instead, Ga reacted with excess N in the environment directly and deposited on the substrate so the growth happens randomly.

In Fig. 2b, when the amount of carrier gas flow was 400 sccm, the density of GaN nanowires reached the highest amount where they also revealed higher length to diameter ratio. The density degraded at either higher or lower amounts of carrier gas flow. The amount of carrier gas flow altered the width of boundary layer which affected the arrival and departure of the precursor gases^36^. The atoms arriving on a solid surface with metal particles, they saw binding sites or potential wells on the substrates. There is a finite probability that atom diffuses along the surface by hoping from well to well; there is also a probability of an atom escaping from the wall (desorption). The absorption and desorption of the precursor elements on the surface of metal particles resulted to different sticking coefficient. For growth, it required that atom diffuse to a step (heterogeneity) before desorption. The ratio of residence time of an atom on the substrate, τ_0_, and diffusion time for an atom to reach a step, τ_D_. The sticking coefficient is

1 $${S}_{c}=\frac{{\tau }_{0}}{{\tau }_{D}}$$

For *S*_*c*_ ≥ 1, an atom will have sufficient time to diffuse to the step and bound to the surface. For *S*_*c*_ < 1, desorption can occur^37^.

Less carrier gas flow resulted to slow and less amount of supply so it reduced the probability/rate of growth, while more carrier gas flow brought the precursor gases traveling faster so that it reduced the sticking of reactants. Besides, taking diffusion boundary layer into consideration where the diffusion was only significant in the boundary layer. Thus there was an optimized range of parameters, based on growth temperature, for stably growing high density and high yield nanowires.

To confirm the above understanding regarding the effects of V/III ratio and carrier gas flow by adjusting the parameters, we demonstrated GaN nanowire growth at different temperature, e.g. 910 °C and 950 °C, with carrier gas flow of 300 sccm, as shown in Fig. 2c. Starting with the obtained optimized V/III ratio and gas flow at 880 °C (V/III = 10–20 and 400 sccm carrier gas flow), there was no growth occurs at neither 910 °C nor 950 °C with the parameters. It was typical that at higher T, there were less Ga and N tend to stay on the substrate, especially for N which was too light with high evaporation rate. So the carrier gas flow should be lowered for allowing elements to stay. The V/III ratio should be increased for getting N-rich environment to enhance axial growth^26^. With the increased V/III ratio (indicated on Fig. 2c) and reduced carrier gas flow rate (from 400 sccm to 300 sccm), Fig. 2c highlighted the successful growth of high density GaN nanowires grown at 910 °C and 950 °C. The lengths of the GaN nanowires were about 20 μm with growth period of 1 min where the growth rate is ~330 nm/s.

Compare the growth at 880 °C at carrier gas flow of 400 sccm and 300 sccm, when changing from 300 sccm to 400 sccm, the diffusion boundary layer was thinner and the probability of element sticking was lower, so that N desorption was more significant at higher carrier gas flow. Based on our understanding discussed above, the N supply must be increased in order to reach the optimized range of nanowire growth. We fixed the amount of Ga and increased the amount of N (V/III ratio goes higher) to obtain high density GaN nanowires. This was in agreement with our experimental data in Fig. 2.

### Photoluminescence of the GaN Nanowires

**Figure 3:**
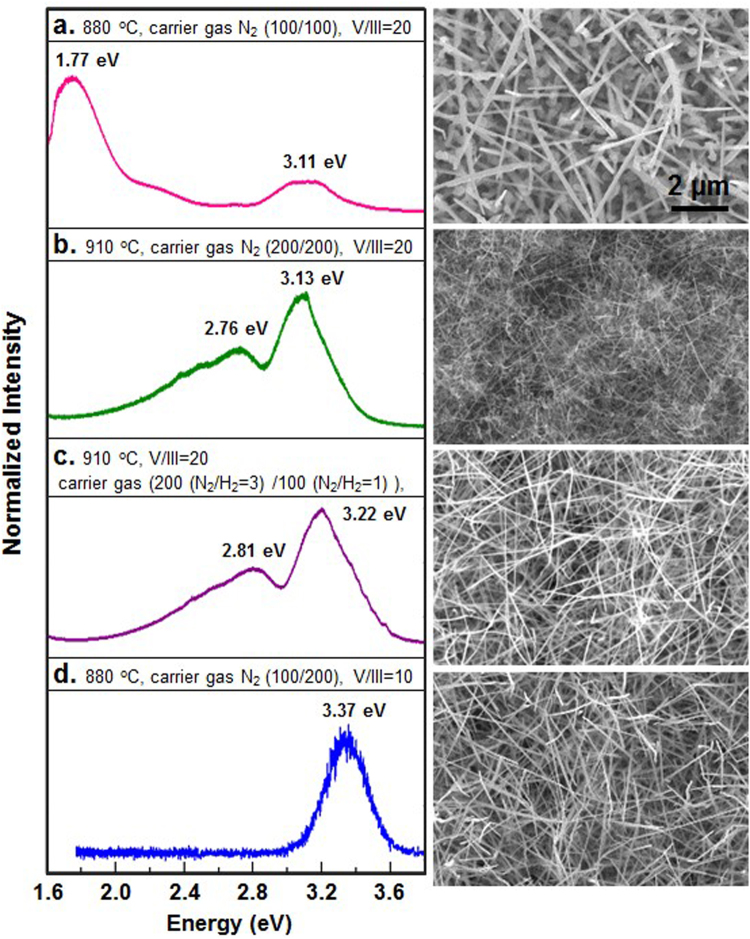
Low temperature photoluminescence (PL) spectra and the corresponding SEM images of GaN nanowires grown with different parameters. (**a**) Red emission from GaN nanowires grown at 880 °C, V/III = 20, and carrier gas of N_2_. (**b**) Near blue band and green band of illumination from GaN nanowires grown at 910 °C, V/III = 20, and carrier gas of N_2_. (**c**) Near blue band and green band of illumination from GaN nanowires grown at 910 °C, V/III = 20, and carrier gas of mixture of N_2_ and H_2_. (**d**) Blue emission from GaN nanowires grown at 880 °C, V/III = 10, and carrier gas of N_2_ with higher flow rate in lower tube. The growth period is 5 min.

Based on our understanding of controlling GaN nanowire growth, here we demonstrated the control of photoluminescence by tuning the defects during growth. Figure 3a showed the GaN nanowires with proper morphology, length, and density; however, they exhibited strong red luminescence, caused by the deep donor of Ga vacancy and O substitutional at N site (~1.1 eV above the valence bound) and the deep acceptor of N vacancy and C substitutional at N site (~0.3 eV below the conduction band)^38–40^, which was not desirable for current innovations in application. We increased the growth temperature and the carrier gas flow, for which the luminescence of nanowires exhibited blue shift as shown in Fig. 3b and showed enhancement in intensity of near blue emission, which indicated the crystals contained fewer defects. The sticking of precursor gases was lower at higher T and higher carrier gas flow resulted to thinner boundary layer; therefore, the nanowire growth rate went down as shown in the corresponding SEM image of Fig. 3b. In addition, Ga vacancy and O interstitial that emitted green/yellow light was yet generated by the unsuccessful growth of nanowires.

To increase the density of nanowires, the carrier gases were adjusted by adding H_2_ to increase the gathering of GaN^37^ and the flow rate of NH_3_ turns down to increase sticking of N. Figure 3c showed the density of nanowires increased with a blue shift in the photoluminescence. We further demonstrated the trend by choosing 880 °C as reaction temperature again but lowered the V/III ratio. We kept the carrier gas to be N_2_ only to reduce H_2_ etching and increased the flow of carrier gas of NH_3_ to keep a reasonable sticking of the molecules. Figure 3d showed the photoluminescence of the nanowires located at 3.37 eV with other emissions negligible, and the density and morphology of nanowires was reasonable as shown in the SEM image. The growth period was longer as 5 min to allow relaxation of vacancy to reduce green/yellow emissions. Compared growth in Fig. 3a and d, lowering V/III ratio made less N-rich environment, lowering carrier gas flow for NH_3_ increased sticking probability of N, and increasing growth period made nanowire grow longer to stabilize Ga vacancy and Si and O interstitials. Note that Ni and Si might diffuse into GaN crystals as Ni interstitials and Si substitutionals. Considering the Stoke Effect^32^, Ni interstitials, Si subsitutionals (donor, 30 meV)^41^, and O interstitials (donor, 29 meV)^41^ result to photoluminescence shift compared with intrinsic GaN so that the main peak of emission slightly shifted from 3.4 eV to 3.37 eV was not surprising. The shift and broader band indicated the existence of Si substitutionals which replaced Ga, stacking faults, O interstitials, and Ni interstitials. In addition, the full width at half maximum of the curves are wider than that of bulk single crystalline GaN because the signals are from samples containing many nanowires with catalysts, the structural defects on the substrate, and contamination caused by the low vacuum growth condition, which could possibly contribute to the shift of emission and the decrease of intensity. Hence, the PL intensity from nanowires is expected to be much lower. Besides, the intensity also relies on the density of nanowires but which is various between samples. Therefore, we focused on the discussion of peak positions but not too much on the intensity change because it was caused by multiple factors.

### Catalyst Adjustment for Growing Long Nanowires

**Figure 4:**
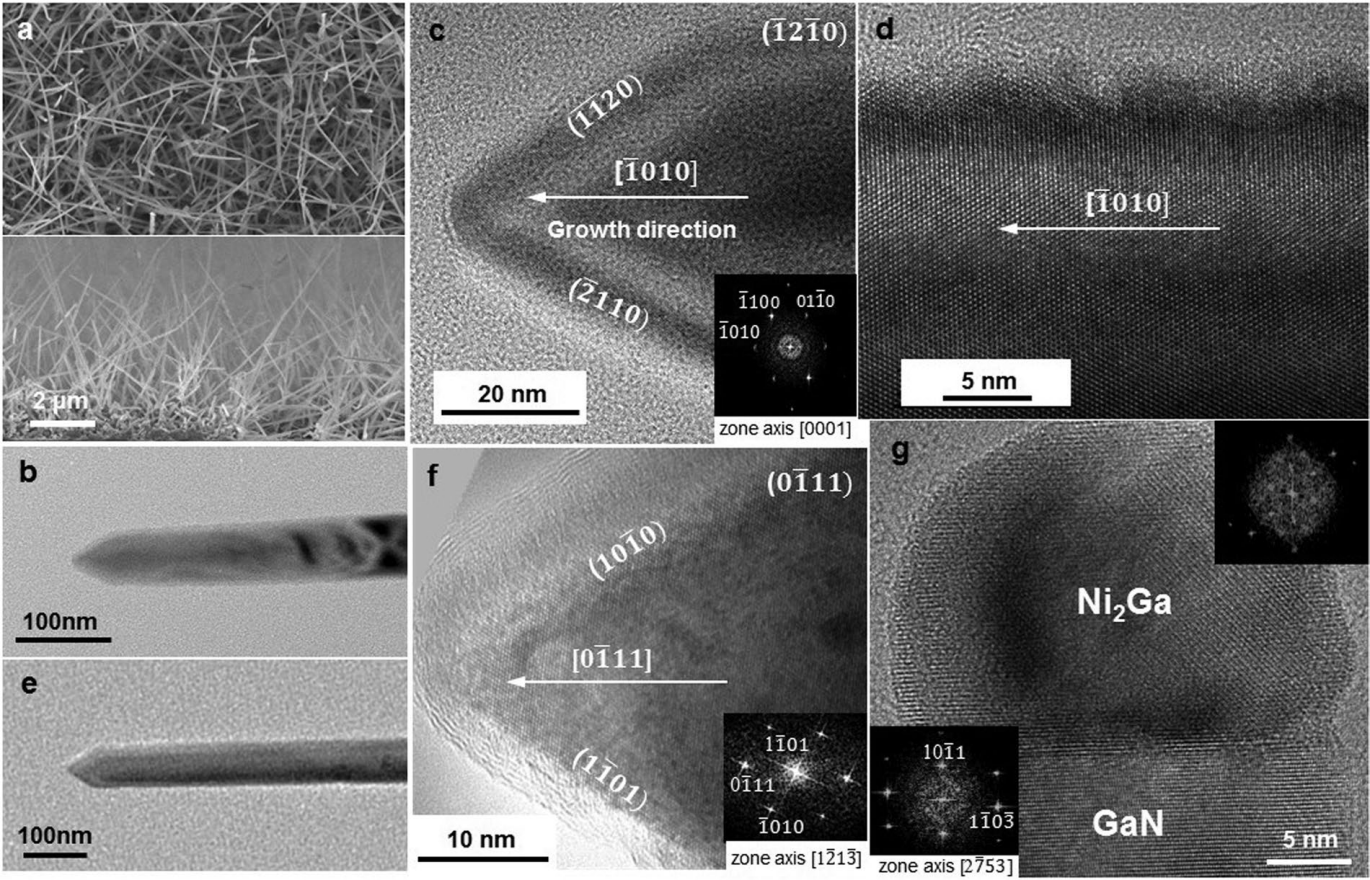
SEM and TEM images of GaN nanowires grown from Ni on Si (111). (**a**) GaN nanowires grown on Si at 880 °C, V/III = 20, and carrier gas flow of 400 sccm. (**b**,**c**,**e**,**f**) Enlarge images of two single GaN nanowires from (**a**), where the catalysts were etched away by HCl during growth. The orientations of the nanowires are <10 $\bar{1}\,$0> and <01 $\bar{1}\,$1>, respectively, and the facets are either m-plane or a-plane. (**d**) A HRTEM image of the body of the GaN nanowire of (**c**). It is single crystalline with smooth sidewall surface. (**g**) A GaN nanowire grown from Ni catalyst at 880 °C, V/III = 10, and carrier gas flow of 300 sccm. The post growth image shows the catalyst particle is Ni_2_Ga and the GaN is single crystalline grew along <10 $\bar{1}\,$1>.

The etching of Ni was not negligible due to the HCl from the flow and the side product of reactions, as discussed in the experimental section. Figure 4a showed the high density of GaN nanowires. In Fig. 4b and e, they showed the nanowires without Ni tips but just GaN facets. Presumably the nanowire growth stopped when Ni was etched away. Two growth orientations were found from the sample to be either <10 $\bar{1}\,$0> or <10 $\bar{1}\,$1> with a-plane or m-plane facets at the tip, as shown in Fig. 4c and f. The variable growth orientation was a typical feature of VSS growth^42^. Ga-polarity dominated where c-plane was stable so that the growth front was along a-plane or m-plane, see example shown in Fig. 4d. Note that Fig. 4c was de-focused to see the facets. We attributed the absence of Ni catalysts to the etching of HCl. To retard the etching process, we lowered the supply of Ga (but kept V/III ratio within the range) and the amount of carrier gas flow. The post growth HRTEM image (Fig. 4g) confirmed that the catalyst was solid Ni_2_Ga. From the phase diagram of Ni and Ga, the catalyst was solid at the growth temperature so that the growth occurred by vapor-solid-solid (VSS) mechanism. The epitaxy of Ni_2_Ga and GaN is shown in Fig. 4g. The growth front of this GaN nanowire was 10 $\bar{1}\,$1.

**Figure 5:**
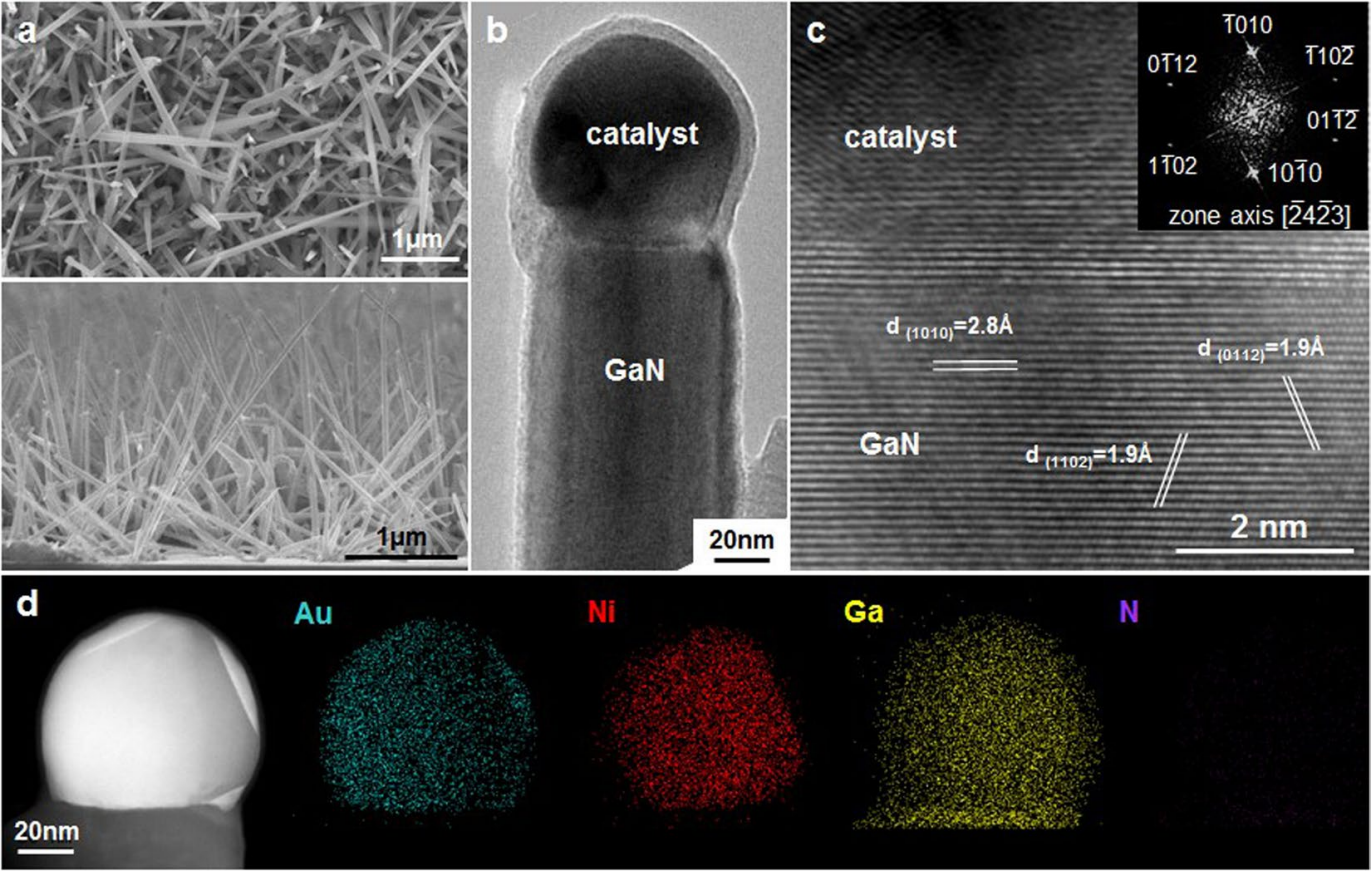
The morphology, orientation and composition of GaN nanowires grown on Si from Au-Ni. (**a**) The plan view and cross-sectional SEM images of the GaN nanowires grown at 880 °C, V/III ratio of 20 and 400 sccm carrier gas flow. (**b**) A TEM image of a GaN nanowire grown from Ni-Au at 850 °C and V/III ratio 40. (**c**) The HRTEM image at the interface of (**b**), where the interface of GaN and catalyst is sharp. The inset is the DP of GaN which confirms the growth direction is <10 $\bar{1}\,$0> and shows the side wall orientation. (**d**) A HAADF STEM image and EDS-STEM mapping of the metal catalyst particle at the tip of another nanowire grown at 850 °C and V/III ratio 40.

Another approach to eliminate the effect of HCl etching was by adding a thin layer of Au on Ni as a protection layer; in addition, Au had higher resistance to oxidation than Ni (The electron negativity of gold is higher than that of nickel which indicates the ionization rate of gold is slower. This attributes to the etch rate of metals) when transferring the samples in air. Figure 5 showed the GaN nanowires grown from Ni-Au under the same growth parameters for growth from Ni (880 °C, V/III = 20, 400 sccm N_2_ carrier gas). Both Ni catalyzed (Figs 1c and 4a) and Ni-Au catalyzed (Figs 1d and 5a) GaN nanowires exhibit single crystallinity, high density (from the plan view and cross section images), and diameter of ~50 nm. It confirmed that the growth from Ni-Au was compatible with that from Ni, but we had better chance to keep the catalysts in position during growth for growing long nanowires. Figure 5b showed a single GaN nanowire with a catalyst particle where the interface of GaN and catalyst is flat. Note that another difference between Ni-catalyzed and Ni-Au catalyzed nanowires is the length obtained at the same growth condition where the Ni catalyzed ones are longer though the length difference can be compromised by adjusting the growth period.

For the long GaN nanowires grown in very short period in HVPE with rapid growth rate, we investigated the crystallinity, orientation, and chemistry of the catalysts. Images in Fig. 2a showed the high density of the straight GaN nanowires. The lengths of these nanowires were about 3–5 μm, which demonstrated the long GaN nanowires were grown in short period of time (1 min in the experiments), which was much faster than conventional growth of GaN nanowires in MOCVD^28,29^. On the lower magnification TEM image (Fig. 5b), the ball-shaped catalyst particle was on the top of the nanowire and the interface of GaN and catalyst was sharp. The corresponding HRTEM image and the diffraction pattern (DP) of nanowire, as shown in Fig. 5c, confirmed the nanowire was single crystalline hexagonal structure and the nanowire was grown along [$\bar{1}\,$010] (see SI). The lattice spacing of ($\bar{1}\,$010) was 2.76 Å, in agreement with the measurement (2.8 Å) from the HRTEM image, indicating the growth front of the GaN nanowire was m-plane wurtize GaN.

The metal catalysts were analyzed on their chemistry by EDS-STEM. Figure 5d showed the high-angle annular dark-field (HAADF) image from another nanowire and EDS-STEM mapping of the catalyst particle showing Au, Ni, and Ga signals. The catalyst particle contained a solid faceted crystal and a thin layer surrounding the crystal. From the mappings in Fig. 5d, Ni accumulated at the center region (the faceted crystal), whereas Au was distributed at the surface. Ga was dispersed in the particle but the signal was a bit stronger at the interface of nanowire and catalyst. There was no N signal because N has high evaporation rate so it was insoluble in the catalyst particle.

**Figure 6:**
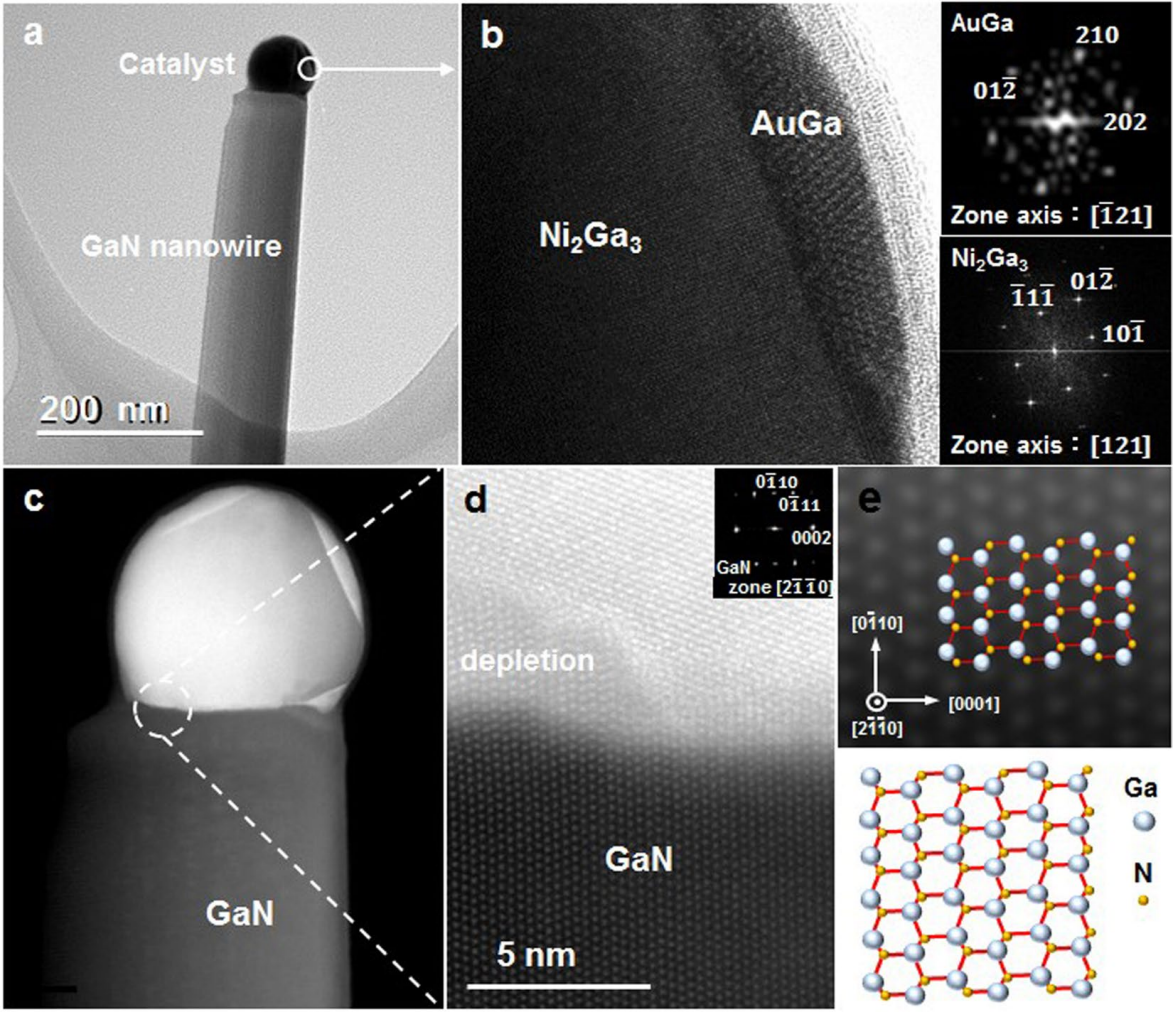
GaN nanowires grown from Ni-Au and the crystallography study in TEM and STEM. (**a**) Post growth TEM image of a GaN nanowire with a catalyst on its tip. (**b**) HRTEM image and DPs of the catalyst particle which confirm the phases in the catalyst is AuGa and Ni_2_Ga_3_. (**c**) ADF-STEM image of the nanowire shown in Fig. 2c. (**d**) A HAADF STEM image of the interface of GaN and the catalyst. The inset is the DP of the GaN. (**e**) The simulation of atomic positions of Ga and N shown on the STEM image. The lower illustration is the atomic arrangement and the bonding viewing in [1 $\bar{1}\,$00] direction.

At the growth temperature of GaN (above 800 °C), from the phase diagrams, Au and Ga formed eutectic liquid and Ni reacted with Ga to form an alloy crystal. The post growth analysis of the AuGa and NiGa phases and the crystallography were shown in Fig. 6. The faceted crystal in the catalyst particle was Ni_2_Ga_3_ and it was surrounded by AuGa (note that at growth T, AuGa is liquid) as shown in Fig. 6b. On the basis of Au-Ga and Ni-Ga phase diagrams, the AuGa was liquid and was expected to encircle the solid Ni_2_Ga_3_ at the growth temperature of 880 °C. Figure 6c showed the ADF-STEM image of the nanowire and the catalyst. The enlarge HAADF image (Fig. 6d) indicated the growth direction was along [0 $\bar{1}\,$10]. The simulation of the atomic positions of Ga and N and the bonding were shown by illustrations on the STEM image as shown in Fig. 6e. The growth orientation were mainly [0 $\bar{1}\,$10] with a few [0 $\bar{1}\,$11], which was consistent with our interpretation on the growth mechanism. The consistency confirmed the self-assembled growth of nanowires. In Fig. 6d, there was a depletion region in the metal at the left corner, which was a typical view of VLS grown nanowires after cooling down due to the solubility change at lower temperature.

The growth fronts of GaN nanowires were mainly m-plane where the inter-planar spacing of each was more similar to Si lattices as one atomic layer of m-plane GaN as 2.76 Å was identical to two bilayers of Si (200) as 2.72 Å. Compared with c-plane growth front of GaN (d = 5.18 Å) on Si (100) or (111), the most obtainable Si wafers, the lattice mismatch is large. So another growth fronts became more favorable in thermodynamic consideration. Besides, nanowires were rarely seen with dislocation and known as effective strain relaxation structure, so GaN growth on Si in nanowire structure via self assembly provided a marvelous approach for growing single crystal GaN with uniform morphology, crystallinity, and orientation.

In addition, the growth of GaN nanowires from Ni catalyst followed classic VSS growth as shown in Fig. 4g, the catalyst is Ni_2_Ga which was solid at the growth temperature. Figure 6b and c show the composition the of catalyst particle as AuGa and Ni_2_Ga_3_ from a Ni-Au catalyzed GaN nanowire. It suggests that the growth followed by both VLS and VSS mechanism as AuGa is liquid and Ni_2_Ga_3_ is solid at the growth temperatures.

## Conclusion

The growth of GaN nanowires on from Ni and Ni-Au catalysts using HVPE was reported. We demonstrated the feasibility for forming the long GaN nanowires within short growth time. The GaN nanowires were single crystalline wurtize along [0 $\bar{1}\,$10] direction with m-plane growth front. The Ni catalyzed GaN nanowires are potentially suitable for integration with CMOS technology. The pure Ni was etched by HCl during growth while adding Au could slow down the effect and enhance the duration of growth to achieve ultra-long GaN nanowires. Using Ni-Au catalyzed method, the longer nanowires are achieved which allows applications on sensors. The Ni-Au catalyst particle contained a faceted Ni_2_Ga_3_ crystal and a liquid AuGa thin layer surrounding Ni_2_Ga_3_ at growth temperature. In addition, the effects of V/III ratio, which changes the surface polarity, and carrier gas flow, which changes the sticking coefficient, were discussed regarding the density and morphology of GaN nanowires. The photoluminescence of red to blue emission from the GaN nanowires have been shown and discussed by controlling the growth parameters as tuning point defects. We suggested an optimized range of parameters of control growth of high density and high crystal quality GaN nanowires, and demonstrated how we tuned the parameters to modify the growth.

## Methods

### Nanowire growth in the HVPE

Si was the substrate for the GaN nanowire growth, which was dipped in a 5% hydrofluoric acid (HF) solution to remove the native oxide before growth experiments. The Si substrates were immediately (within 5 min) transferred into an electron gun evaporation chamber for metal deposition at 2 × 10^−5^ Torr. Ni of 5 nm thick was deposited on Si and/or followed by deposition of 1 nm thick Au, where Au forms islands rather than a continuous film due to the very small addition from deposition, for protection from oxidation and etching of Ni. GaN nanowires were grown in a horizontal HVPE^43^, where the base pressure is 700 Torr, via vapor-liquid-solid or vapor-solid-solid growth mechanisms. The precursor gases were ammonia (NH_3_) and gallium chloride (GaCl) forming by flowing HCl gas diluted with nitrogen through molten Ga at 850 °C. GaN then grew when the two precursor gases, GaCl (as the supply of Ga) and NH_3_ (as the supply of N), met and reacted near the samples at 650–950 °C. High purity N_2_, as the carrier gas, is flowed and stabilized before the injection of precursor gases. The flow rate of N_2_ was 2–3 times larger than the flow rate of the precursor gases. The growth period for most samples reported here was 1 min except a few ones grew longer in time. The V/III ratio, defined as the ratio of the amounts of gas flow of NH_3_ and HCl, was ranged from 10 to 100 during the process.

The precursor gas gallium chloride (GaCl) was formed by flowing HCl gas diluted with nitrogen through molten Ga at 850 °C as

2 $$2{{\rm{HCl}}}_{({\rm{g}})}+2{{\rm{Ga}}}_{({\rm{melt}})}\leftrightarrow 2{{\rm{GaCl}}}_{({\rm{g}})}+{{\rm{H}}}_{2({\rm{g}})}$$

GaN then grew when GaCl and NH_3_ reacted at 650–950 °C as

3 $${{\rm{GaCl}}}_{({\rm{g}})}+{{\rm{NH}}}_{3({\rm{g}})}\leftrightarrow {{\rm{GaN}}}_{({\rm{s}})}+{{\rm{HCl}}}_{({\rm{g}})}+{{\rm{H}}}_{2({\rm{g}})}$$

There were additional reactions involved as listed below.

4 $${{\rm{HCl}}}_{({\rm{g}})}+{{\rm{NH}}}_{3({\rm{g}})}\to {{\rm{NH}}}_{4}{{\rm{Cl}}}_{({\rm{s}})}$$

5 $${{\rm{GaCl}}}_{({\rm{g}})}+2{{\rm{HCl}}}_{({\rm{g}})}\to {{\rm{GaCl}}}_{3({\rm{g}})}+{{\rm{H}}}_{2(g)}$$

6 $${{\rm{GaCl}}}_{3({\rm{g}})}+{{\rm{NH}}}_{3({\rm{g}})}\to {{\rm{GaN}}}_{({\rm{s}})}+3{{\rm{HCl}}}_{({\rm{g}})}$$

The side product HCl was also involved in the etching process. There is always H_2_ gas generated which affect growth.

### Characterization

The GaN nanowires were investigated using SU-8010 scanning electron microscopy (SEM) and JEOL-ARM200F scanning transmission electron microscopy (TEM and STEM).

### Photoluminescence measurement

The PL spectra of the GaN nanowires were measured at 10 K using a He-Cd laser with wavelength of 325 nm.

## Electronic supplementary material

Supplementary Information
