# Supplementary material for: Controlling bottom-up rapid growth of single crystalline gallium nitride nanowires on silicon
Source: Sci Rep. 2017 Dec 20;7:17942. doi: 10.1038/s41598-017-17980-0 (PMC5738410; doi:10.1038/s41598-017-17980-0)
Supplement: Supplementary file 1 — Supplementary Information [file 41598_2017_17980_MOESM1_ESM.pdf]

Supporting Information

Controlling bottom-up rapid growth of single crystalline gallium nitride nanowires on silicon

Ko-Li Wu,[†] *Yi Chou,*[†] *Chang-Chou Su,*[†] *Chih-Chaing Yang,*[†] *Wei-I Lee,*[†] *Yi-Chia Chou*[*,†]

[†]Department of Electrophysics, National Chiao Tung Univeristy, Hisnchu 300, Taiwan

1. X-Ray diffraction and EDS Analysis

The texture of the sample grown with GaN nanowires was examined using x-ray diffraction as shown in Fig. S1a. The 1100 (m-plane), 1101, 1120 (a-plane) orientations were dominate, which was in agreement with our crystallographic analysis. The EDX result showed Si and O was detectable where Si could replace Ga and O could replace N. Such substitutional defects generated near blue emission were as shown in Fig. 3 in the main text.

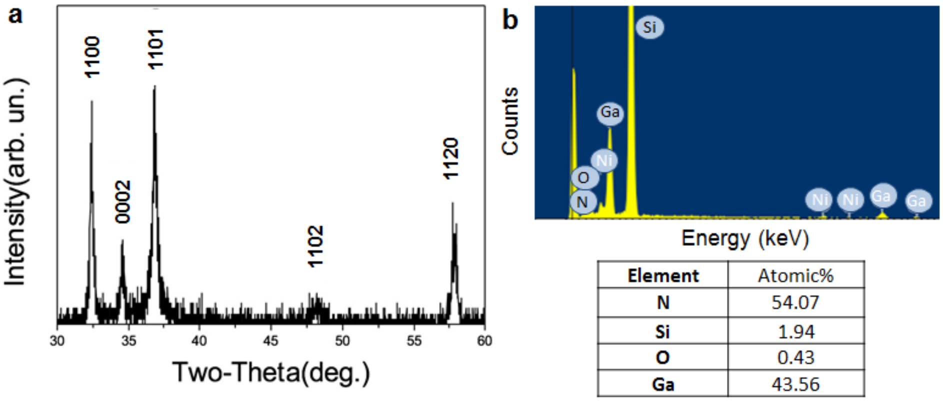

Element	Atomic%
N	54.07
Si	1.94
O	0.43
Ga	43.56

Figure S1. Examination of GaN nanowires grown on Si for 1 min at 880 °C with V/III ratios of 20 under N_2 carrier gas flow rate at 400 sccm. (a) X-ray diffraction spectrum. (b) EDX in SEM of a single GaN nanowire.

2. Microscopic details of the GaN nanowires

Figure S2 showed the mass production of the GaN nanowires from Au-Ni reported in the main as shown in Figure 5 and 6. Every single nanowire was straight with smooth surface and a catalyst particle sitting at the top.

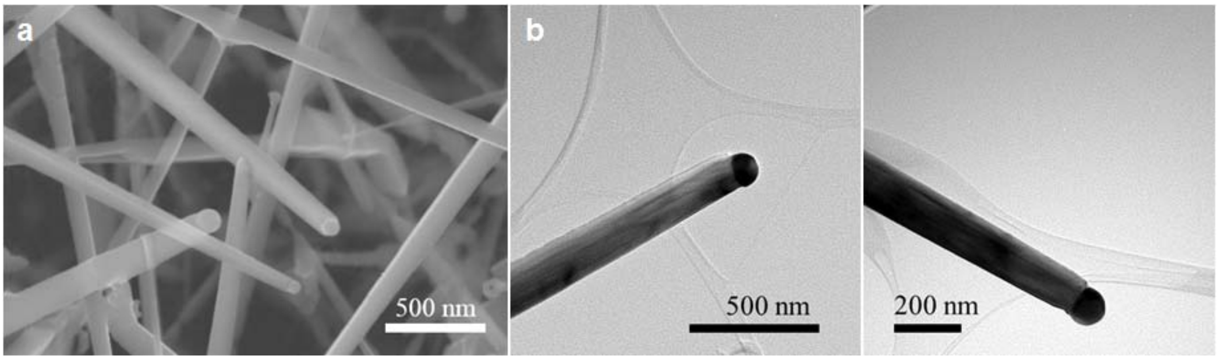

Figure S2. Electron microscopic images of GaN nanowires grown from Au-Ni. (a) SEM images showing the plan view of the wafer with grown GaN nanowires with catalyst particle on the top. (b) TEM images of three single GaN nanowire with catalyst where it is on the top of each.

To show the details of the depletion region as shown in Figure 6d in the main text, a HRTEM image and the DPs were shown and discussed in Figure S3a. We confirmed again the nanowire was single crystalline GaN and the catalyst particle was crystalline. There was a depletion region at the triple junction of nanowire, catalyst, and air, where the corresponding DP was in the upper right inset. The depletion region contained GaN which was known as caused from the solubility change in catalyst during cooling process so that GaN precipitate when temperature went down.

Figure S4 showed the TEM and HRTEM images of the sidewall and near-core of the GaN nanowire. The atomic structure and diffraction pattern from the HRTEM image confirm that the body of the GaN nanowire exhibit single crystallinity with wurtzite structure.

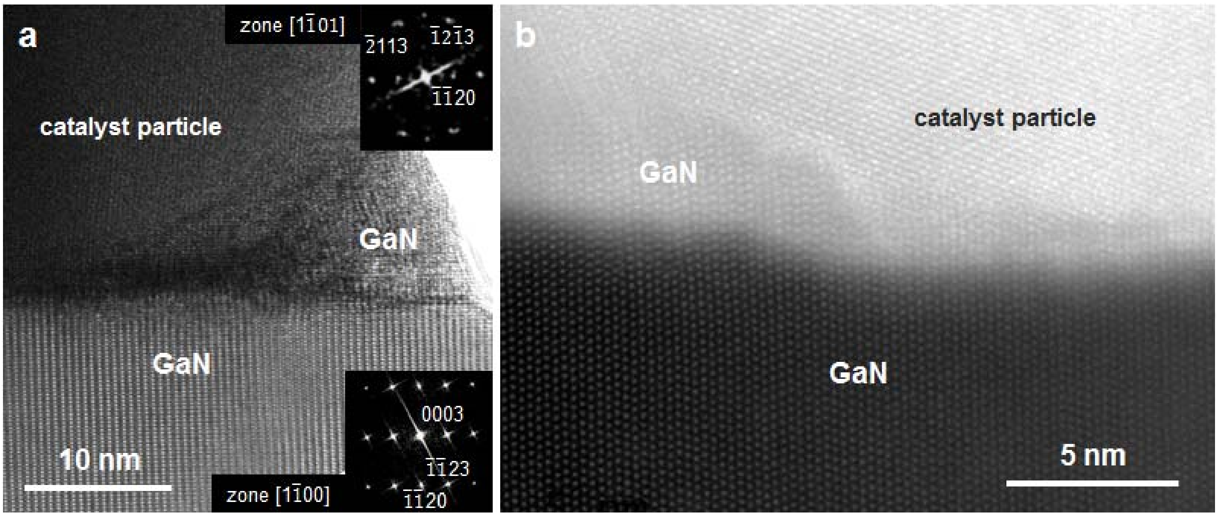

Figure S3. HRTEM (a) and ADF-STEM (b) images of the triple point of nanowire, catalyst, and air. (a) The lower right inset is the DP of nanowire and the upper right one is the DP of the depletion region. The nanowire is single crystalline GaN and the depletion region is GaN. (b) The image with wider view of Fig 6d. It shows the GaN is single crystalline.

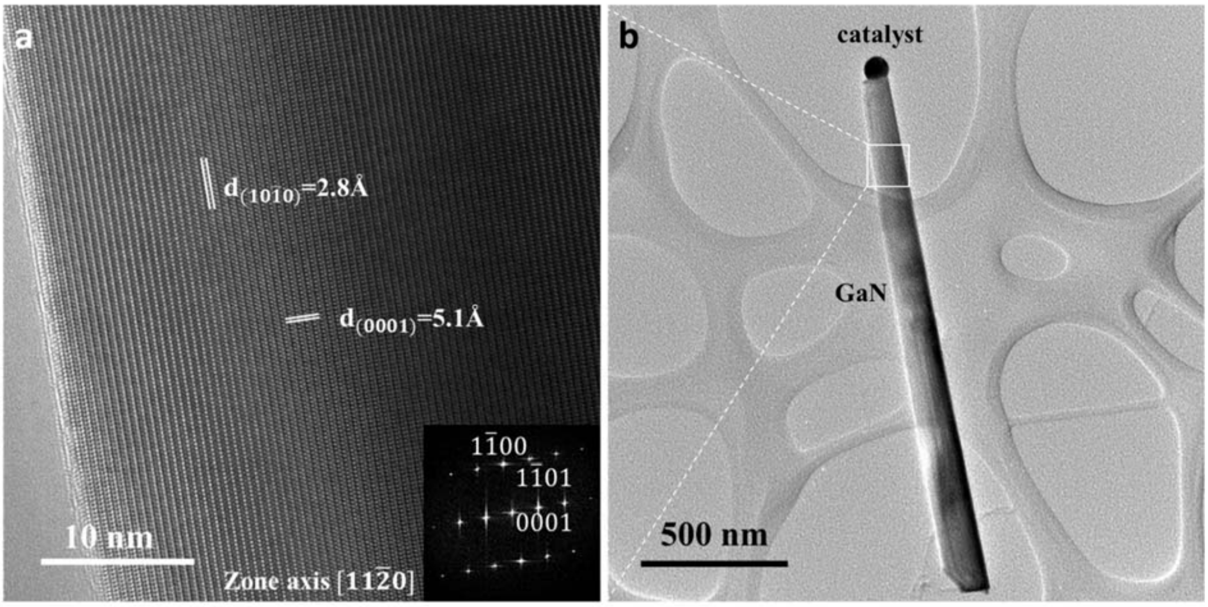

Figure S4. Images from the sidewall and near-core of a GaN nanowire. (a) The HRTEM image of GaN nanowire at the sidewall, and inset is DP of nanowire. (b) The full view of the transferred GaN nanowire.

3. Effect of growth temperature on the nanowire growth

Fig. S5 2 showed the temperature dependence of the nanowire morphology and density. The nanowires exhibit nanowire form at 880 ^{o}C while others show irregular nanorods or kinked structures.

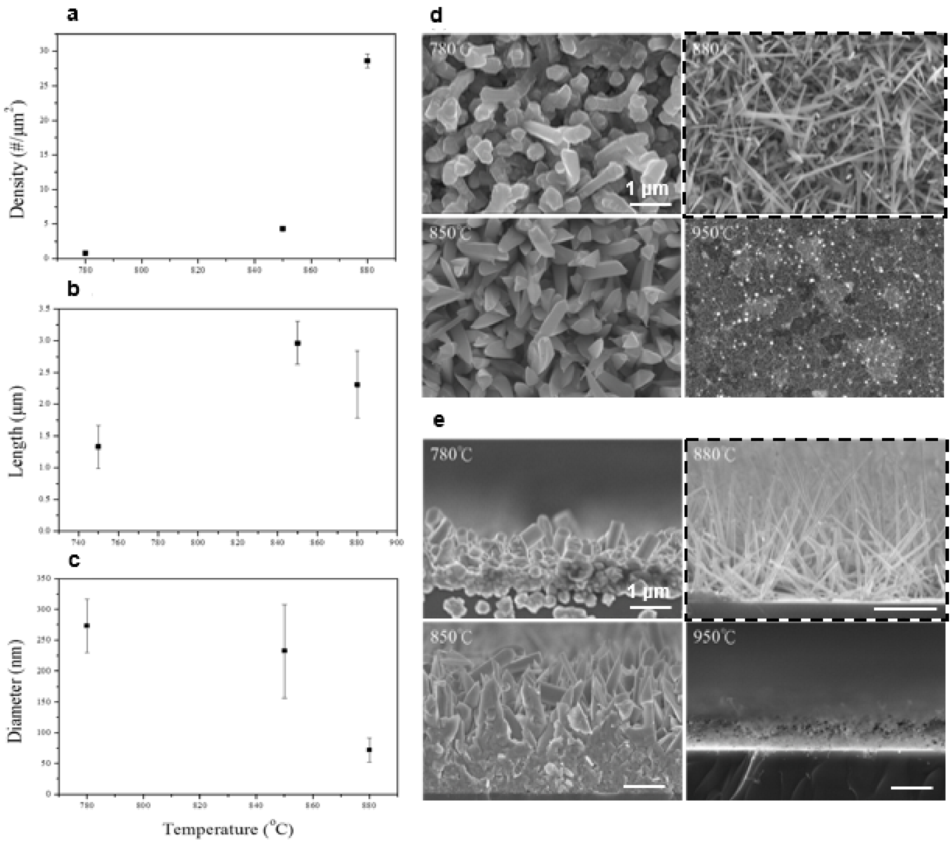

Figure S5. Effects of temperature on the growth of GaN nanowires on Si (111). (a-c) The density, length, and diameter of nanowires grown at Ⅴ/Ⅲ ratio of 20 and carrier gas flow of 400 sccm at different V/III ratios. (d, e) Plan view and cross section SEM images of the GaN nanowires grown at different T. The best grown nanowires is highlighted by the dashed frame where the growth rate is 38±6 nm/s.

4. Effect of cooling rate

The step of cooling is critical to keep the grown materials with its crystallinity. The growth temperature of GaN nanowires was above 800 ^{o}C where the catalyst was relatively mobile. If the sample was kept in the chamber when stopping growing, the reaction could continue with the residual heat in the chamber. So the cooling rate must be fast enough to eliminate the undesired structure formation with residual heat and gases. At the temperature lower than growth temperature, but still high enough for nucleation and growth of GaN, the supersaturation of product from the catalyst kept changing with T where the stoichiometry in the catalyst might also change with the slowly decreasing T. Figure 1e is one of the examples without quick cooling, where last grown structure at inconstant T (shown near the interface of catalyst and nanowire) contains defect such as stacking faults.

In addition, the catalyst was less stable without flowing gases. The avoid catalyst diffusion and keep the nanowires as they stopped growing, the cooling process was investigated. When we stopped nanowire growth, the heating was turned down and the NH_3 was kept flowing to maintain the stability of the catalyst during cooling down. To avoid the impact from the residual heat in the chamber, we moved the samples out as quickly as we could when the temperature was handle-able. Fig. S3b confirmed that the GaN nanowire near the interface was single crystalline and the stacking faults after careful cooling treatment were much less than that shown in Fig. 1e.
